# Supplementary material for: Effect of biologic treatments on growth in children with juvenile idiopathic arthritis: A systematic review
Source: PLoS One. 2025 May 28;20(5):e0324440. doi: 10.1371/journal.pone.0324440 (PMC12118834; doi:10.1371/journal.pone.0324440)
Supplement: S1 Table — (DOCX) [file pone.0324440.s001.docx]

| Supplementary table S1: Main characteristics and results of the selected studies aiming to assess the effect of biologics on growth in juvenile idiopathic arthritis patients: | | | | | | |
| --- | --- | --- | --- | --- | --- | --- |
| First author (ref)  Year of publication  Country | **Study design/**  **Characteristics of the population studied** | **Inclusion, exclusion criteria** | **Biologic treatment,** **Duration of the follow-up period** | **Growth assessment criteria** | **Main outcomes** | **Conclusion**  **of the authors** |
| Schmeling et al.  2003  Germany | Cohort study  (N=7) | Inclusion  JIA patients with growth delay (HSDS <-2), treated with Etanercept.  Exclusion  Patients treated with GH. | .Etanercept twice weekly at  a dosage of 0.4 mg/kg subcutaneously  Follow-up  3 years | .HSDS  .Height velocity (cm/year)  .Serum levels of IGF and IGFBP-3 | .The growth rate of all patients increased significantly after Etanercept therapy.  .This effect was most pronounced in the first year of  treatment.  .Pre-treatment levels of IGF-I and IGFBP-3 were low, but within the normal range in all patients.  .IGF-I and IGFBP-3 levels significantly increased after biologic treatment.  .The increase in IGF-1 levels was not only seen in  patients who had undergone puberty but also in prepubertal children.  .An inverse correlation of IGF-1 serum levels to CRP was demonstrated. | .Etanercept which control disease activity led to an increase growth rate with catch-up growth. |
| Tynjala P et al.  2006  Finland | Cohort study (n=71) | Inclusion  JIA treated with biological agents for more than 1 year  Exclusion  -Patients> 15 years at the initiation of anti-TNF treatment  -Patients with less than 12 months of anti-TNF treatment;  -Patients ongoing or previous GH therapy. | .Etanercept (0.4 mg/kg twice weekly  Subcutaneously): 43 patients.  .Infliximab (80 to  200 mg intravenously every 6 to 8 weeks): 28 patients.  Follow-up  Two years before and 2 years after the initiation of  anti TNF-alpha treatment. | .Height and weight  .z score (SD)  .HSDS  .Growth velocity  .Growth rate (cm/year)  .Height adjusted relative weight (%)  .BMI  .Skeletal maturation  (assessed before and 2 years after  anti-TNF). | .There was a significance increase in the mean growth velocity between the two-year follow up periods before and after the initiation of TNF alpha inhibitors treatment (76% of patients improved their growth velocity on  anti-TNF treatment. Of these, 78% had delayed growth).  .No differences between the patients receiving etanercept or infliximab treatment in the change of growth parameters.  .Patients with delayed growth improved their growth velocity significantly during biological treatment. | .The growth rate before the anti-TNF treatment was the strongest  predictor for the observed change in the growth velocity.  .The change in inflammatory activity  remained a significant predictor of the growth velocity, even after glucocorticoids were taken into account. |
| Vojvodich al.  2007  Sweden | Cohort study  (N=31) | Inclusion  -Prepubertal or early/midpubertal  Patients  -Patients adherent to treatment for at least 1 year  Exclusion  -Treatment stopped within the first year due to insufficient clinical improvement or adverse events  -Concomitant treatment  with GH  -Advanced puberty  -Missing data before etanercept treatment. | Etanercept  Follow-up  1 year | .Growth velocity  .Adjusted Parental HSDS | .Improvement of growth velocity: 85 and 72% of pubertal patients respectively  .The growth improvement in the pubertal group did not reach statistical significance.  .The improvement in growth was negatively correlated with the need of intraarticular glucocorticoids (a marker for disease activity). | .Etanercept improved growth in a majority of patients with JIA,  independently of the pubertal growth spurt. |
| Biliau et al.  2010  Belgium | Cohort study (n=16) | Inclusion  Etanercept treatment started at a dose of at least 10 mg/m²/week, for at least 3 months prior to the enrollment date  Exclusion  Patients with major concurrent medical  conditions | Etanercept  Follow-up  18 months | .HSDS  .Growth velocity  .BMD at lumbar spine, z score (SD)  .Body composition (% lean body mass,  % fat mass and % bone mineral content) | .A mild progressive increase in HSDS was throughout the study in both groups, but at none of the time points did the comparison with baseline reach statistical significance.  .A significant increase was noted in lumbar Z-scores was noted in Etanercept group whereas the increase in MTX-treated patients was not statistically significant  .A significant increase BMC was noted after biological treatment.  .In the etanercept +MTX group, but not in the MTX group, the lean mass: fat mass ratio increased, with a statistically significant increase.  .Etanercept effects on growth are associated with a  reduction of systemic IL-6 and an increase in systemic  OPG levels | .Clinical efficacy of etanercept in MTX-refractory  polyarticular JIA patients is associated with rapidly instituted catch-up growth.  .MTX-refractory polyarticular JIA patients responding to Etanercept  show rapidly improved bone mineralization and body composition. |
| Giannini et al.  2010  Ohio | Cohort study  (n=594) | Inclusion  Patients aged 2–18 years with selected categories of JIA treated with Etanercept within 6 months  Exclusion  Pregnant or nursing patients, anti-TNF antibody, anti-CD4  antibody, or IL-2 diphtheria fusion protein treatment,  anti-TNF agent other than etanercept, cyclophosphamide, or other biologic agents, serious medical conditions, or a history of drug and/or  alcohol abuse; or any malignancies within the 5 years | Etanercept  twice weekly at a dose of 0.4 mg/kg or once  weekly at a dose of 0.8 mg/kg (maximum dose 50 mg) with or without combination with MTX  Follow-up  3 years | - Height (cm)  -Height (percentile)  -Weight (kg)  -Weight (percentile)  -BMI (kg/m2) | .The mean height percentile was significantly increased from baseline for etanercept at year 3 and for etanercept plus MTX at years 1, 2, and 3.  .No consistent effect of disease control on height was observed for any of the study treatments (MTX, Etanercept).  .Normal development (as assessed  by Tanner staging scores) did not appear to be adversely affected in any of the study treatments. | .Etanercept with or without MTX improved growth rates  in children with JIA. |
| Uettwiller F et al.  2013  France | Cohort study (n=100) | Inclusion  -Prepubertal or early pubertal JIA patients  at biologic treatment onset  -Patients under  biologic therapy for at least 6 months  Exclusion  -Postpubertal patients (Tanner staging > 3) at biologic therapy onset  -Patients with a joint deformity, associated medical condition that could modify their growth,  GH therapy, insufficient retrospective data | .TNF-alpha inhibitors: (n=51)  .Anti-IL1: (n=28)  (7 patients received anakinra then canakinumab and 3 received only canakinumab)  .Anti-IL-6: (n=12)  .CTLA-4Ig (abatacept): (n=9)  -Follow-up  6 months after biological treatment onset. | - Height and weight, z score (SD)  -Patient target height (SD)***  - HSDS corrected for target height  - Growth velocity | - A low mean growth velocity at the initiation of biologic therapy and an improvement under biologic treatment.  -In patients who had developed growth retardation before the onset of biologic therapy, it persisted at the latest follow up.  - Patients treated with >1 biologic agent and patients with systemic JIA had significantly lower changes in the HSDS on biologic treatment than other patients.  - Steroid usage and active disease were not significantly associated with lower changer in HSDS after biologic treatment. | .Biologic therapy has a positive effect on changes in the  height SDS.  .Biologic therapy  might be insufficient to restore normal growth in patients who do not adequately  respond to their first biologic agent.  .Early initiation of  biologic therapy and selecting an optimal first biologic agent may be important options for improving the final  height of patients with JIA. |
| Miyamae et al.  2013  Japan | Cohort study  (n=45) | Inclusion  Systemic JIA: Boys <10 years and girls <8 years  Exclusion  -TCZ treatment for at least 3 years in the phase III and the following extension studies  -Concurrent DMARDs or immunosuppressive therapies other than corticosteroids | TCZ 8 mg/kg/2 weeks  Follow-up:  .2 years for 28 patients  .4 years for 17 patients | -HSDS (for 45 patients)  - Height velocity (cm/year): for 28 patients  - Height velocity extended (cm/year): 17 patients | .A clear inverse correlation between baseline HSDS and disease duration  .No clear correlation between baseline corticosteroid dose and HSDS.  .Patients with less than median corticosteroid exposure had significant improvement in Δ HSDS during the study.  .Mean HSDS significantly improved from the 1 year before initiation of TCZ treatment to 1 year after.  .The only factor to show a correlation with improvement in height velocity was reduction in corticosteroid exposure after initiation of TCZ treatment. | .It is difficult to evaluate independently how much the effect of TCZ contributes to the improvement as compared with  the effect of reducing corticosteroid exposure in this study. |
| Shafferman et al.  2014  US | Case control  Study group  (n=167)  Control group (n=37) | Inclusion  -At least 1 clinic visit with BMI measurement prior to the first initiation of TNF-alpha inhibitors therapy; and at least 1 clinic  visit following a minimum of 365 days of uninterrupted therapy after first initiation of TNF-alpha inhibitors. /  Exclusion  -Patients who initiated TNF-alpha inhibitors treatment before the age of 2 years,  -Patients with systemic JIA  or inflammatory bowel disease | .TNF-alpha inhibitors: all patients  Follow-up:  2.8 years. | -BMI (kg/m2)  -BMI (SD)  -BMI category (normal, overweight, obese)  -Weight (kg)  -Height (cm) | .TNF-alpha inhibitors had a statistically significant increase in mean BMI and BMI z-score during the study period, that was not significantly different from the increase observed in the comparator cohort.  .There was no significant change in the proportions of overweight and obese children in patients treat with TNF-alpha inhibitors compared to baseline or compared to the change in the comparator cohort. | .The use of TNF-alpha inhibitors in JIA patients is not associated with an  excessive weight gain. |
| Świdrowska et al.  2015  Poland | Retrospective  (n=21) | Inclusion  JIA with CID treated with biologics  Exclusion  Patients who had received GH therapy | .Etanercept (0.8 mg/kg/week: (n=10)  .Adalimumab (40mg/ 2 weeks): (n=8)  .Tocilizumab (8 to 12 mg/kg/ 2weeks or 10 mg/kg/month): (n=5)  .Golimumab (30mg/m2 body surface): (n=2)  Follow-up:  12 months up to 2 years. | -HSDS | -Improvement of the growth velocity was seen in 15 patients with JIA (71.4%).  -No significant differences between the particular therapies.  -In around half of the children with growth rate improvement the daily dose of corticosteroids could be significantly reduced. | .Biological agents restore growth velocity not only by inflammation  inhibition, but also through limiting corticosteroids daily doses. |
| Kearsley-Fleet et al.  2015  UK | Cohort study  (n=191) | Inclusion  Patients with complete height data recorded at baseline, 1 and  2 years | .Etanercept: All patients  Follow-up  2 years | -Height, z score (SD)  -HSDS  -BMI | .Etanercept therapy was associated with an improvement in height z-score over the first 2 years of therapy.  .Mean height z-scores of patients with systemic  arthritis remains the lowest after 2 years.  .Children with JIA not receiving corticosteroids experienced greater height improvement.  .Disease activity was weakly associated with improvement in growth following Etanercept therapy. | Etanercept improved growth rates in children with JIA, but the mean height z-score at 2 years remains lower than that of the reference population. |
| De Benedetti et al.  2015  43 centers members of the PRINTO and the PRCSG | RCT  (n=83) | Inclusion  Age between 2 years and 17 years, persistent disease (≥6-month duration) with inadequate response to previous non steroidal anti-inflammatory drugs and corticosteroids.  Exclusion  Previous treatment with GH, patients with near epiphyseal closure, patients who had reached Tanner stage 5 at the end of the first year. | TCZ: 12 mg/kg for body weight <30 kg; 8 mg/kg for body weight ≥30 kg  Follow-up  1 year for 83 patients and 2 years for 55 patients. | - HSDS  -BMI (SDS)  -Height velocity (cm/year)  - Serum IGF-1 levels  - Serum level of OC  -Serum level of CTX-1 | .Baseline HSDS was negatively correlated with duration of systemic JIA.  .In 43 patients, Pretreatment height velocity was below normal in 88% of patients (87.5%  of females and 89% of males).  .A Significant catch-up in height velocity during TCZ treatment.  .There was no important change in BMI over time.  .At baseline, IGF-1 levels were significantly lower than normal levels for age.  .IGF-1 levels significantly increased after TCZ treatment (very close to normal levels).  .Baseline serum levels of OC and of CTX-1  were related to CRP levels consistent with the link between inflammation and bone turnover.  .During the initial 6 months of treatment, there  was a rapid increase in the average height velocity.  .The rapid increase in height velocity was  accompanied by a marked improvement in disease activity and by a marked decrease in the  mean dose of corticosteroids.  .Baseline age, average glucocorticoid dose during year 1, disease activity at year 1, and  baseline height SDS were significant predictors of height velocity during the first year of treatment. | .TCZ treatment therapy markers in patients with systemic JIA was associated with positive effects on growth velocity, GH/IGF-1 axis and soluble bone markers.  .There is a need for the tapering of glucocorticoid dose to exploit the beneficial effects of  tocilizumab on the growing skeleton.  .This tapering should be balanced with the need to maintain  adequate disease control, particularly during the early phase of treatment. |
| Bharucha et al.  2018  the PRINTO and the PRCSG | RCT  (n=187) | Inclusion  Age between 2 years and 17 years, patients with polyarticular JIA or extended oligoarticular JIA, disease duration of 6 months at least, inadequate response or intolerance to MTX,  5 active joints at least at baseline, stable dose of corticosteroids ≤ 0.2 mg/kg/day prednisone or 10 mg/day  for at least 4 weeks before.  Exclusion  Previous treatment with GH. | TCZ: randomly assigned to 8 or 10 mg/kg for body weight < 30 kg; 8 mg/kg for body weight≥ 30 kg) for 16 weeks.  Follow-up  2 years with an open-label extension through Week 104. | -HSDS  -Height velocity (cm/year) (n=103) | .Baseline HSDS was not related to age or disease duration.  .Mean height SDS increased significantly in patients with Tanner stage ≤ 3 at baseline after 2 years of TCZ treatment.  .There was no evident difference in 2-year height velocity in the 81 patients who received placebo for a mean duration of about 18 weeks  in Part 2 of the trial compared with those who received TCZ continuously. | Patients with polyarticular JIA or extended oligoarticular JIA experienced adequate or  improved growth over 2 years of treatment with TCZ, particularly  those whose growth impairment at baseline was greatest. |

JIA: Juvenile idiopathic arthritis; SDS: Standard-deviation-score; TNF: Tumor-necrosis-factor; CID: chronic inflammatory diseases; GH: Growth hormone; IGF-1: insulin-like growth factor-1; IGFBP-3: insulin-like growth factor (IGF)-binding protein-3; OPG: osteoprotegerin; TCZ: Tocilizumab

HSDS: Height SDS defined as observed height minus mean height for age divided by SD, where SD was the standard deviation for the normal population of the same chronological age and sex; BMD=Bone mineral density; BMI= Body Mass Index; BMC=Bone mineral content; PRINTO: Paediatric Rheumatology International Trials Organisation; PRCSG: Pediatric Rheumatology Collaborative Study Group, HSDS: Height SDS defined as observed height minus mean height for age divided by SD

*: ΔHSDS≥ 0: catch up growth and ΔHSDS<0: impaired growth; Height adjusted relative weight (%): the ratio of weight for height (W/H) in (kg/cm) to the mean W/H in the normal population of the same calendar age and sex.

** determined according the Greulich-Pyle method in relation to the measurements in normal Finnish children

*** Patient target height: calculated using Tanner’s formula

+ Growth velocity: Δ HSDS during follow up

° Height SDS was subtracted by the mid-parental target (the sum of the father’s and mother’s height plus 13 cm in a boy or minus 13 cm in a girl, all divided by 2) height SDS
