# Supplementary material for: Effect of biologic treatments on growth in children with juvenile idiopathic arthritis: A systematic review
Source: PLoS One. 2025 May 28;20(5):e0324440. doi: 10.1371/journal.pone.0324440 (PMC12118834; doi:10.1371/journal.pone.0324440)

**Supplementary Table 2a: Quality of the studies according to MINORS**

|  | **Score** | **Interpretation** |
| --- | --- | --- |
| Gianini et al, 2020 | 10 | Moderate |
| Schmelling et al, 2003 | 8 | low |
| Kearsley-Fleet et al, 2015 | 9 | moderate |
| Miyamae et al, 2014 | 11 | moderate |
| Uettwiller et al, 2014 | 6 | low |
| Billiau et al, 2019 | 9 | moderate |
| Tynjala et al, 2006 | 12 | moderate |
| Vojvodich et al, 2007 | 7 | low |
| Shafferman et al, 2013 | 9 | moderate |

**Supplementary Table 2b: Quality of the studies according to CONSORT**

|  | **Score** |
| --- | --- |
| De Benedetti et al (2015) | 13 |
| Bharucha et al (2018) | 16 |

**Risk of biais assessment**

**Supplementary Table 3a:** Risk of bias assessment according to the Newcastle Ottawa Scale (NOS) for the cohort studies

|  | **Selection** | **Comparability** | **Outcome** | **Score** |
| --- | --- | --- | --- | --- |
| Gianini et al, 2020 | *** | * | *** | 7 |
| Schmelling et al, 2003 | ** | * | ** | 5 |
| Kearsley-Fleet et al, 2015 | **** | * | ** | 7 |
| Miyamae et al, 2014 | *** | * | *** | 7 |
| Uettwiller et al, 2014 | *** | ** | *** | 8 |
| Billiau et al, 2019 | *** | * | *** | 7 |
| Tynjala et al, 2006 | *** | * | ** | 6 |
| Vojvodich et al, 2007 | ** | * | ** | 5 |

**Supplementary Table 3b:** Risk of bias assessment according to the Newcastle Ottawa Scale (NOS) for the case control

|  | **Selection** | **Comparability** | **Exposure** | **Score** |
| --- | --- | --- | --- | --- |
| Shafferman et al, 2013 | *** | * | ** | 6 |

**Supplementary Table 3c:** Risk of bias domains accoding to the revised Cochrane risk-of-bias tool for randomized trials (RoB 2)

|  | **D1** | **D2** | **D3** | **D4** | **D5** | **Overall** |
| --- | --- | --- | --- | --- | --- | --- |
| **De Benedetti et al (2015)** | 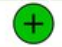 | 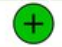 | 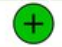 | 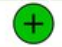 | 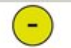 | 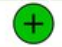 |
| **Bharucha et al (2018)** | 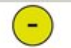 | 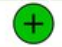 | 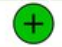 | 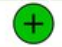 | 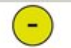 | 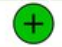 |


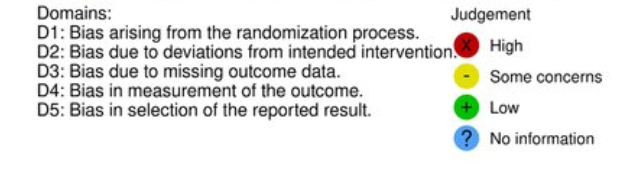

Supplement: S2 Table — (DOCX) [file pone.0324440.s002.docx]
