# Supplementary material for: Effect of biologic treatments on growth in children with juvenile idiopathic arthritis: A systematic review
Source: PLoS One. 2025 May 28;20(5):e0324440. doi: 10.1371/journal.pone.0324440 (PMC12118834; doi:10.1371/journal.pone.0324440)
Supplement: Attachment — Submitted filename: response to reviewers.docx [file pone.0324440.s006.docx]

**Dear reviewers, thank you for your pertinent remarks. We hope that we have adequately responded to all your comments.**

**Journal comments**

1. Please ensure that your manuscript meets PLOS ONE's style requirements, including those for file naming.

The manuscript was modified according to plos one requirements.

2. Thank you for stating the following in your Competing Interests section:  
"NO authors have competing interests"

The statement was added as requested

2. Please include a caption for figure 1.

A caption was added for figure 1

3. Please include captions for your Supporting Information files at the end of your manuscript, and update any in-text citations to match accordingly.

Captions for supporting informations were added at the end of the manuscript.

Additional Editor Comments:

All the revision was marked in done in revision mode and highlighted in green.

This systematic review provides updated insights into the impact of biologic treatments on growth in pediatric patients with juvenile idiopathic arthritis (JIA). While an addition to scientific literature, I believe that a major revision of the write up is warranted. Please see my comments below:

1- Please keep all percentage points referenced to the same decimal points for consistency. For example, in the introduction, authors write “10.4% in children with polyarticular disease to 41% of patients with the system form.”

This was modified accordingly in the paragraphs as well as in tables.

2- In the introduction, is the main focus on the effects of biologic treatments for growth “retardation” in children with JIA? I think this statement could use a little clarification for the reader.

Indeed, the main focus was to explore the effect of biologic therapy in children with juvenile idiopathic arthritis on growth. Specifically, the question is whether the biologics improve growth in JIA independently of the existence of an initial growth retardation. This clarification was added as requested in the abstract and in the introduction. (Line 77)

3- In the first paragraph of the methods section, can you add more detail to how disputes were resolved? In cases where consensus could not be reached, was a third party consulted to arbitrate the decision?

As requested, we added these clarification were added. “Extracted data from each study was scrutinized independently by both investigators (Dr MY and Dr BAH). They used a pilot-tested extraction form. In cases where consensus could not be reached, a third party (Dr BH) was consulted to resolve disagreements.” (Line 116-Line 121)

4- Instead of stating “The two first authors of this systematic review agreed on the articles to be included in this paper” I would advise to specify the two authors involved as you did in the first paragraph of the methods section.

Thank you for your remark, this was added as requested. (Line 92)
5- For the inclusion criteria, what is meant by “full papers”? Does this mean “full-text papers?” To ensure a robust selection process, please review that studies included were required to meet the following criteria: (1) publication in peer-reviewed journals, (2) relevance to the research question, and (3) availability of sufficient data for extraction in addition to your other already included study specific criteria.

Thank you for your remark. Indeed, we meant full-texts. This was added in the selection criteria section as requested. (Line 97-Line 100)

6- Please also include the date of inception in the first sentence for clarity.

The date of inception was added as requested. (Line 96)

7- In the inclusion criteria section, please review the numbered points on studies selected, they skip number 3.

Thank you for your remark. This was rectified as requested (Line 105)

8- In the inclusion criteria section, this sentence “Studies assessing clinical and/or biological and/or radiological growth parameters before and after biological therapy” could be revised for better clarity.

Thank you for your remark. This sentence was revised to avoid redundancy and enable better comprehension. "Studies evaluating clinical, biological, and radiological growth parameters before and after biological therapy." (Line 104)

9- For number 1 of the exclusion criteria, please add an example of “other autoimmune diseases that may compromise the growth velocity”.

Thank your for remark. An example was added as requested. (Line 108)
10- Please revise the last sentence of the exclusion criteria to improve clarity and maintain a formal tone. Specifically, change "Besides" to "Additionally" and rephrase "not in compliance with the systematic review purpose" to "that did not align with the objectives of the systematic review."

Thank you for your remark. This revision was added to improve clarity for the reader. (Line 113-114)

11- In the Data Extraction and Quality Assessment section, please revise the following sentence “The extracted data included the main methodological characteristics of the articles: study data (year of publication, country, study design, number of included subjects, mean age of included subjects, inclusion and exclusion criteria, duration of the followup)” for better clarity and readability. Specifically, change "included the main methodological characteristics of the articles: study data (year of publication, country, study design, number of included subjects, mean age of included subjects, inclusion and exclusion criteria, duration of the followup)" to "included the main methodological characteristics of the articles, such as the year of publication, country, study design, number of subjects, mean age of subjects, inclusion and exclusion criteria, and duration of follow-up."

Thank you for your remark. This was modified accordingly.(Line 119-121)

12- In the Data Extraction and Quality Assessment section, please fix the spelling of “judgement”

The spelling was corrected as requested. (Line 121)
13- In the Data Extraction and Quality Assessment section, what does “high quality” mean? Please expand on this.

The quality of the studies was identified through Newcastle Ottawa Scale (NOS) for the cohort and case control studies and the revised Cochrane risk-of-bias tool for randomized trials (RoB 2). To avoid confusion for the reader, the sentence has been deleted. The evaluation was the quality of the studies was also pertinent to perfume metaanalysis, which was not the case. Indeed, we could not perform comparison as the methods of evaluation differed among studies.
14- In the results, this statement: “The studies, published between 2003 (17) and 2018 (23),” makes it seem as though there were 17 studies selected in 2003, and 23 studies selected in 2018. Since you state “between”, this is not clear. Please revise for clarity and readability.

The numbers 17,23 referred to first (2003) and last article published (2018). To avoid confusion, the references were added at the end of the sentence. (Line 131)
15- In the results section, “The” does not have to be capitalized before “United States” or “United Kingdom”
Thank you for your remark. This was corrected accordingly. (Line 133)
16- For this statement “Notably, two studies (24,25) were multicenter trials conducted by the members of the Paediatric Rheumatology International Trials Organisation (PRINTO) (24) and the Pediatric Rheumatology Collaborative Study Group (PRCSG) (27): The TENDER trial and the CHERISH trial (28, 29) . The applied designs were the cohort study (13,17–20,23), the open-label non randomized trial (11), the open-label randomized placebo-controlled trial (12,24,25), the case control study (21) and the retrospective study (22)”, I am getting confused on which studies were the “two notable studies.” Please revise the statement for better clarity and to clearly identify the references for the notable studies and study designs. For example, change "Notably, two studies (24,25) were multicenter trials conducted by the members of the Paediatric Rheumatology International Trials Organisation (PRINTO) (24) and the Pediatric Rheumatology Collaborative Study Group (PRCSG) (27): The TENDER trial and the CHERISH trial (28, 29)" to "Notably, two studies, the TENDER trial and the CHERISH trial, were multicenter trials conducted by members of the Paediatric Rheumatology International Trials Organisation (PRINTO) (24) and the Pediatric Rheumatology Collaborative Study Group (PRCSG) (27), respectively."
Thank you for your pertinent remark and for this suggestion. The sentence was reformulated accordingly. (Line 134-137)
17- For the results section, when reporting on patient characteristics, the placing of the references is confusing. Please revise for readability.
Regarding the paragraph of patient characteristics, the references were replaced at the end of the sentences to avoid confusion for the reader. (Line 149-Line 154)

18- Table 1 could be improved for better clarity and readability. Ensure consistent formatting and alignment of columns and rows, and use clear, concise column headers without abbreviations. Separate different types of data into subsections or separate tables, and include footnotes to explain any abbreviations or unique terms. Highlight key data points with bold or italicized text, and add summary statistics like means and medians. Use visual aids to distinguish groups or highlight differences, and maintain consistency in units of measurement throughout the table. Remove redundant information and provide a clear caption summarizing the table’s content along with any relevant notes.

The formatting of the table 1 was revised as requested. We added a column for extremes. We added mean and medians when provided. We maintained the same units and added footnotes when required to facilitate the comprehension of the table.

19- In the results 1.4 Growth Assessment Criteria, this section would read easier in paragraph format for a scientific journal. This follows into sections 1.5 and 1.6, which read more like a paper outline instead of a finished manuscript.

The results were modified as requested. (Line 177-Line 230)
20- Please add a review of all biologics that were included in the studies reviewed prior to the discussion section.
The distribution of the different biologics according to the mechanism of action was recorded in the manuscript as requested. (Line 170-174)
21- The discussion section provides a comprehensive summary of the findings, emphasizing the positive impact of biologic therapies on growth in children with JIA. However, it would benefit from more structured presentation and clearer emphasis on key points.

We organized the discussion into distinct paragraphs with clearer presentation. We also emphasized the most important points specifically the positive impact of biologic therapies on growth.

22- Consider organizing the discussion into distinct paragraphs that separately address the main findings, the implications of these findings, and the limitations of the study. Highlight the most significant improvements in growth parameters, such as HSDS and growth velocity, and discuss their clinical relevance.

We organized the discussion into distinct paragraphs with clearer presentation. We also emphasized the most important points specifically the positive impact of biologic therapies on growth. We also discussed the clinical relevance of such results. (Line 234-291)
23- In the discussion section - while the potential mechanisms and differences based on factors like puberty stage and JIA subtype are well-covered, it would be helpful to further elaborate on the practical implications for treatment strategies and patient management.
Thank you pertinent remark. As requested, we highlighted practical implications on such findings. Specifically, how initiating biologic therapy early in the disease course, is beneficial for patients presenting with growth delays or high disease activity. (Line 241-280)
24- Lastly, explicitly state the limitations of the review, such as heterogeneity in growth assessment criteria and the observational nature of some studies, to provide a balanced perspective.

As requested, the limitations of the review were highlighted. (Line 281-294)
