# Supplementary material for: Effect of biologic treatments on growth in children with juvenile idiopathic arthritis: A systematic review
Source: PLoS One. 2025 May 28;20(5):e0324440. doi: 10.1371/journal.pone.0324440 (PMC12118834; doi:10.1371/journal.pone.0324440)
Supplement: Attachment — Submitted filename: respond to rev 2.0.docx [file pone.0324440.s007.docx]

Dear Editor,

We would like to thank you for the opportunity to resubmit a revised copy of this manuscript. We would also like to take this opportunity to express our thanks to the reviewers for the positive feedback and helpful comments for correction. We have revised the manuscript accordingly and provided a point-by-point response below.

We hope the revised manuscript will be suitable for publication in your journal.

Best regards

D. MAKHLOUF YASMINE

Answers to reviewers:

1- **In the PRISMA flowchart, you specify that originally 97 records were screened for this study. Please provide a numbered table of all those 97 studies identified in the literature search, including those that were excluded from the analyses. For every excluded study, the table should list the reason(s) for exclusion:**

We provided, as requested, a numbered table of the 89 studies identified in the literature search and which remained after removing duplicates (8 duplicates). Please see the attached table

2- **Please also update the methods section of your manuscript with an explanation of how missing data were handled:**

Missing data were handled by first attempting to contact study authors for additional information. If data remained unavailable, we mentioned it in the tables. One of the main reasons for not performing a metanalysis in the heterogeneity in the studies. We also assessed the risk of bias due to missing data using using the revised Cochrane risk of bias tool for randomized trials, the Newcaslte Ottawa Scale for the case control studies and for the cohort studies. Excluded data were then excluded from the analysis.

3- **Thank you for providing a table showing the completed risk of bias and quality/certainty assessments for 11 of the included studies. Please update this information to reflect the risk of bias and quality/certainty assessment of all 12 included studies:**

We provided the risk of bias and quality assessments all the studies as requested. All the data is provided in supplementary table 2 and 3.
